# Supplementary material for: CLEESE: An open-source audio-transformation toolbox for data-driven experiments in speech and music cognition
Source: PLoS One. 2019 Apr 4;14(4):e0205943. doi: 10.1371/journal.pone.0205943 (PMC6448843; doi:10.1371/journal.pone.0205943)
Supplement: S2 Code — (HTML) [file pone.0205943.s007.html]

analysis


## CLEESE: An open-source audio-transformation toolbox for data-driven experiments in speech and music cognition

This Python notebook contains code used in the paper : Burred, J.J., Ponsot, E., Goupil, L., Liuni, M. & Aucouturier, JJ. (2019) *CLEESE: An open-source audio-transformation toolbox for data-driven experiments in speech and music cognition* , to illustrate the analysis of reverse-correlation data from the paper's case-study #1 and #2.
Uses the CLEESE toolbox, available from http://forumnet.ircam.fr/product/cleese.   
**Author:** JJ Aucouturier (CNRS/IRCAM, 2018)

In [ ]:

```
%matplotlib inline
import matplotlib.pyplot as plt
import glob, os
import numpy as np
from numpy.polynomial import polynomial as P
import re, csv
import pandas as pd
from math import sqrt
import seaborn as sns
from numpy.polynomial import polynomial as P
from scipy import stats
from scipy.stats import linregress
sns.set_style("whitegrid")
```

## Case-study 1 : Speech intonation

We give here a proof of concept of how to use CLEESE in a reverse-correlation experiment to uncover what exact pitch contour drives participants' categorization of an utterance as interrogative or declarative.

**Stimuli:** : One male speaker recorded a 426ms utterance of the French word 'vraiment' ('really'), which can be experienced either as a one-word statement or question. We used CLEESE to artificially manipulate the pitch contour of the recording. First, the original pitch contour (mean pitch = 105Hz) was artificially flattened to constant pitch. Then, we added/subtracted a constant pitch gain ($\pm$ 20 cents, equating to $\pm$ 1 fifth of a semitone) to create the 'high-' or 'low-pitch' versions presented in each trial. Finally, we added Gaussian 'pitch noise' (i.e. pitch-shifting) to the contour by sampling pitch values at 6 successive time-points, using a normal distribution (SD = 70 cents; clipped at +/- 2.2 SD), linearly interpolated between time-points.

**Procedure** : 700 pairs of randomly-manipulated voices were presented to each of N=5 observers (male: 3, M=22.5yo), all native French speakers with self-reported normal hearing. Participants listened to a pair of two randomly-modulated voices and were asked which of the two versions was most interrogative. Inter-stimulus interval in each trial was 500 ms, and inter-trial interval was 1s.

**Analysis** : We compute a first-order temporal kernel (i.e., a 7-points vector) for each participant, as the mean pitch contour of the voices classified as interrogative minus the mean pitch contour of the voices classified as non-interrogative. Kernels are then normalized by dividing them by the absolute sum of their values and then averaged over all participants for visualization. We use a one-way repeated-measures ANOVA on the temporal kernels to test for an effect of segment on pitch shift, and posthocs computed using Bonferroni-corrected Tukey tests.

#### Load data

In [3]:

```
result_dir = 'data/interrogative/results/'
result_files = glob.glob(result_dir+"results_subj*.csv")
results = all_results_df = pd.DataFrame()
for result_file in result_files: 
    participant_results = pd.read_csv(result_file, sep = ',', decimal='.', header='infer')
    results = results.append(participant_results, ignore_index=True)
```

#### Computes 1st Order Kernels (from Ponsot et al., 2018)

In [4]:

```
# first-order temporal KernelAudio (5-points vector) computed for each subject as the
# mean(Pitch of voices classified as interrogative) - (mean P of voices classified as non-interrogative)
segment_mean_pitch = results.groupby(['subj','segment','response'])['pitch'].mean().reset_index()
positives = segment_mean_pitch.loc[segment_mean_pitch["response"] == True].reset_index()
negatives = segment_mean_pitch.loc[segment_mean_pitch["response"] == False].reset_index()
kernels = pd.merge(positives, negatives, on=["subj","segment"])
kernels["kernel_pitch"] = kernels["pitch_x"] - kernels["pitch_y"]

# KernelAudios were then normalized in each condition by dividing them 
# by the sum of their absolute values and then averaged in each task
kernels["square_pitch"] = kernels["kernel_pitch"]**2
for_norm = kernels.groupby(['subj'])['square_pitch'].mean().reset_index()
kernels = pd.merge(kernels, for_norm, on=["subj"])
kernels["norm_pitch"] = kernels["kernel_pitch"]/np.sqrt(kernels["square_pitch_y"])
```

#### Plot the kernels

In [5]:

```
fig = plt.figure(figsize=(10,5))
plt.style.use('default')
plt.subplots_adjust(wspace = 0.2, hspace = 0.8)

ax1 = fig.add_subplot(121)
plt.style.use('default')
ax = sns.tsplot(time="segment", value="norm_pitch", unit="subj", data=kernels.reset_index(), ci=95, ax=ax1)
plt.xlabel("Segment")
plt.ylabel("Normalized Pitch Kernel (a.u.)")
plt.plot([0,6],[0,0],color='k',linestyle = ':')

for subj in 1+np.arange(0,5) : 
    ax2 = fig.add_subplot(5,4,4*subj-1)
    subj_data = kernels[kernels['subj']==subj]
    plt.plot(subj_data['segment'], subj_data['norm_pitch'])
    plt.plot([0,6],[0,0],color='k',linestyle = ':')
    _=plt.xlim([0,6])
    _=plt.ylim([kernels.norm_pitch.min(),kernels.norm_pitch.max()])
    plt.title("Subject "+str(subj))
    ax2.title.set_fontsize(8)
    
plt.subplot(5,4,4*subj-1)
plt.xlabel("Segment")
```

```
C:\Python27\lib\site-packages\seaborn\timeseries.py:183: UserWarning: The `tsplot` function is deprecated and will be removed in a future release. Please update your code to use the new `lineplot` function.
  warnings.warn(msg, UserWarning)
C:\Python27\lib\site-packages\scipy\stats\stats.py:1706: FutureWarning: Using a non-tuple sequence for multidimensional indexing is deprecated; use `arr[tuple(seq)]` instead of `arr[seq]`. In the future this will be interpreted as an array index, `arr[np.array(seq)]`, which will result either in an error or a different result.
  return np.add.reduce(sorted[indexer] * weights, axis=axis) / sumval
```

Out[5]:

```
<matplotlib.text.Text at 0x10b1fd30>
```

In [6]:

```
g = sns.factorplot(x="segment", y="norm_pitch", col = "subj", data=kernels.reset_index(), 
                  capsize = 0.1, ci = 95, whis=np.inf, aspect = 1, size = 2.5)
```

```
C:\Python27\lib\site-packages\seaborn\categorical.py:3666: UserWarning: The `factorplot` function has been renamed to `catplot`. The original name will be removed in a future release. Please update your code. Note that the default `kind` in `factorplot` (`'point'`) has changed `'strip'` in `catplot`.
  warnings.warn(msg)
C:\Python27\lib\site-packages\seaborn\categorical.py:3672: UserWarning: The `size` paramter has been renamed to `height`; please update your code.
  warnings.warn(msg, UserWarning)
```

In [7]:

```
from rpy2.robjects import r,pandas2ri
pandas2ri.activate()
from rpy2.robjects.packages import importr
base = importr('base')
ez = importr('ez')
df=pandas2ri.py2ri(kernels)

r_anova = r("""
library(ez)
function(df) {
    segment <- as.ordered(df$segment)
    subject <- as.factor(df$subj)
    norm_pitch <- as.numeric(df$norm_pitch)
    data_anova <- data.frame(subject,segment,norm_pitch)
    ezANOVA(
        data=data_anova,
        dv=norm_pitch,
        wid=subject,
        within=segment,
        detailed=TRUE)
}
""")
print r_anova(df)

# returns multcmp posthocs
r_lme = r("""
library(nlme)
library(multcomp)
function(df) {
    segment <- as.ordered(df$segment)
    subject <- as.factor(df$subj)
    norm_pitch <- as.numeric(df$norm_pitch)
    data_anova <- data.frame(subject,segment,norm_pitch)
    Lme.mod <- lme(norm_pitch ~ segment, random = ~1 | subject, data = data_anova)
    #summary(Lme.mod)
    #anova(Lme.mod)
    summary(glht(Lme.mod, linfct=mcp(segment = "Tukey"), test = adjusted(type = "bonferroni")))
}
""")
print r_lme(df)
```

```
$ANOVA

       Effect DFn DFd       SSn       SSd         F            p p<.05

1 (Intercept)   1   4  7.947374 0.3000443 105.94933 5.024700e-04     *

2     segment   6  24 24.067187 2.6853948  35.84901 7.840077e-11     *

        ges

1 0.7269286

2 0.8896433


	 Simultaneous Tests for General Linear Hypotheses


Multiple Comparisons of Means: Tukey Contrasts


Fit: lme.formula(fixed = norm_pitch ~ segment, data = data_anova, 

    random = ~1 | subject)


Linear Hypotheses:

           Estimate Std. Error z value Pr(>|z|)    

1 - 0 == 0  0.05428    0.20652   0.263   1.0000    

2 - 0 == 0 -0.19967    0.20652  -0.967   0.9611    

3 - 0 == 0 -0.06437    0.20652  -0.312   0.9999    

4 - 0 == 0  0.27082    0.20652   1.311   0.8469    

5 - 0 == 0  2.38530    0.20652  11.550    <0.01 ***

6 - 0 == 0  0.44022    0.20652   2.132   0.3336    

2 - 1 == 0 -0.25395    0.20652  -1.230   0.8828    

3 - 1 == 0 -0.11865    0.20652  -0.575   0.9975    

4 - 1 == 0  0.21654    0.20652   1.049   0.9426    

5 - 1 == 0  2.33103    0.20652  11.287    <0.01 ***

6 - 1 == 0  0.38594    0.20652   1.869   0.5014    

3 - 2 == 0  0.13530    0.20652   0.655   0.9949    

4 - 2 == 0  0.47049    0.20652   2.278   0.2547    

5 - 2 == 0  2.58498    0.20652  12.517    <0.01 ***

6 - 2 == 0  0.63989    0.20652   3.099   0.0323 *  

4 - 3 == 0  0.33519    0.20652   1.623   0.6673    

5 - 3 == 0  2.44968    0.20652  11.862    <0.01 ***

6 - 3 == 0  0.50459    0.20652   2.443   0.1809    

5 - 4 == 0  2.11448    0.20652  10.239    <0.01 ***

6 - 4 == 0  0.16940    0.20652   0.820   0.9830    

6 - 5 == 0 -1.94508    0.20652  -9.419    <0.01 ***

---

Signif. codes:  0 '***' 0.001 '**' 0.01 '*' 0.05 '.' 0.1 ' ' 1

(Adjusted p values reported -- single-step method)
```

## Case-study 2: musical rhythm¶

Here, we give a proof of concept of how to use CLEESE in a reverse-correlation experiment to uncover what temporal contour drives participants' judgement of a rhythmically competent/accurate rendition of the well-known song *Happy birthday*.

**Stimuli** : One female singer recorded a *a capella* rendering of the first phrase of the French folk song 'Joyeux anniversaire' (translation of English song 'Happy birthday to you'). We used CLEESE to artificially manipulate the timing of the recording by stretching it between different breakpoints. First, we manually identified the time onset of each sung note in the phrase, and use these positions as breakpoints. Second, the original temporal contour was artificially flattened (i.e. all notes were stretched/compressed to have identical duration), while preserving the original pitch of each note. The duration of this final stimulus was 3203ms. Then, we added Gaussian 'temporal noise' (i.e. time-stretching) to each note by sampling stretch values at 6 successive time-points, using a normal distribution centered at 1 (SD = 0.4; clipped at +/- 1.6 SD), using a square BPF with a transition time of 0.1 s. The resulting stimuli were therefore sung variants of the same melody, with the original pitch class, but random rhythm (and overall duration).

**Procedure**:
Pairs of these randomly-manipulated sung phrases were presented to N=12 observers (male: 6, M=22yo), all native French speakers with self-reported normal hearing. Five participants had previous musical training (more than 12 years of instrumental practice) and were therefore considered as musicians, the other seven participants had no musical training and were considered as non-musicians. Participants listened to a pair of two randomly-modulated phrases and were asked which of the two versions was best sung/performed. Inter-stimulus interval in each trial was 500 ms, and inter-trial interval was 1s. Each participant was presented with a total of 313 trials. There were 280 different trials and the last block of 33 trials was repeated twice (in the same order) to estimate the percentage of agreement. After the test, all participants complete the {rhythm, melody, rythm-melody} subtests of the Profile of Music Perception Skills (PROMS, Law & Zentner, 2012), in order to quantify their melodic and rhythmic perceptual abilities.

**Analysis** :
A first-order temporal kernel (i.e., a 6-points vector) is computed for each participant, as the mean stretch contour of the phrases classified as 'good performances' minus the mean stretch contour of the phrases classified as 'bad performances'. Because stretch factors are ratio, the kernel is in fact computed in the log stretch domain, as mean log t+ - mean log t-, where t+ and t- are the contours of selected and non-selected trials, resp. Kernels are then normalized by dividing them by the absolute sum of their values and then averaged over all participants. A one-way repeated-measures ANOVA is conducted on the temporal kernels to test for an effect of segment on time-stretch, and posthocs are computed using Bonferroni-corrected Tukey tests.

#### load data¶

In [8]:

```
result_dir = 'data/birthday/results/'
result_files = glob.glob(result_dir+"results_subj*.csv")
results = all_results_df = pd.DataFrame()
for result_file in result_files: 
    participant_results = pd.read_csv(result_file, sep = ',', decimal='.', header='infer')
    results = results.append(participant_results, ignore_index=True)
```

All participants did the same number of trials

In [9]:

```
trial_count = results.groupby(['subj'])['trial'].count()/12
trial_count
```

Out[9]:

```
subj
1     320
2     320
3     320
4     320
5     320
6     320
7     320
8     320
9     320
10    320
11    320
12    320
Name: trial, dtype: float64
```

#### compute kernels¶

In [10]:

```
# first-order temporal KernelAudio (5-points vector) computed for each subject as the
# mean(Pitch of voices classified as interrogative) - (mean P of voices classified as non-interrogative)
results['log_stretch']=np.log(results['stretch'])
segment_mean_stretch = results.groupby(['subj','segment','response'])['log_stretch'].mean().reset_index()
positives = segment_mean_stretch.loc[segment_mean_stretch["response"] == True].reset_index()
negatives = segment_mean_stretch.loc[segment_mean_stretch["response"] == False].reset_index()
kernels = pd.merge(positives, negatives, on=["subj","segment"])
kernels["kernel_stretch"] = np.exp(kernels["log_stretch_x"] - kernels["log_stretch_y"])

# KernelAudios were then normalized in each condition by dividing them 
# by the sum of their absolute values and then averaged in each task
kernels["abs_stretch"] = abs(kernels["kernel_stretch"])
for_norm = kernels.groupby(['subj'])['abs_stretch'].sum().reset_index()
kernels = pd.merge(kernels, for_norm, on=["subj"])
kernels["norm_stretch"] = kernels["kernel_stretch"]/kernels["abs_stretch_y"]
```

In [11]:

```
# first-order temporal KernelAudio (5-points vector) computed for each subject as the
# mean(Pitch of voices classified as interrogative) - (mean P of voices classified as non-interrogative)
results['log_stretch']=np.log(results['stretch'])
segment_mean_stretch = results.groupby(['subj','segment','response'])['log_stretch'].mean().reset_index()
positives = segment_mean_stretch.loc[segment_mean_stretch["response"] == True].reset_index()
negatives = segment_mean_stretch.loc[segment_mean_stretch["response"] == False].reset_index()
kernels = pd.merge(positives, negatives, on=["subj","segment"])
kernels["kernel_stretch"] = kernels["log_stretch_x"] - kernels["log_stretch_y"]

# KernelAudios were then normalized in each condition by dividing them 
# by the sum of their absolute values and then averaged in each task
kernels["abs_stretch"] = abs(kernels["kernel_stretch"])
for_norm = kernels.groupby(['subj'])['abs_stretch'].sum().reset_index()
kernels = pd.merge(kernels, for_norm, on=["subj"])
kernels["norm_stretch"] = kernels["kernel_stretch"]/kernels["abs_stretch_y"]
#kernels["norm_stretch"] = np.exp(100*kernels["norm_stretch"])
```

In [12]:

```
proms_results = pd.read_csv(result_dir+'/proms.csv', sep = ',', decimal='.', header='infer')
kernels = pd.merge(kernels, proms_results, on="subj")
```

#### plot the kernels¶

In [13]:

```
#order by 
g = sns.factorplot(x="segment", y="norm_stretch", col = "subj", row="musician", data=kernels.reset_index(), 
                  capsize = 0.1, ci = 95, whis=np.inf, aspect = 1, size = 2.5)
```

In [14]:

```
plt.figure(figsize=(10,5))
plt.style.use('default')
plt.style.use('default')
plt.subplots_adjust(wspace = 0.25)

plt.subplot(121)
kernels['musician'] = (kernels['musician']==1)
score = np.log(np.array([0.75,0.25,1,1,1,2]))

segment_mean = kernels.groupby(['segment'])['norm_stretch'].mean()
kernel_range = segment_mean.max()-segment_mean.min()
score_range = score.max() - score.min()
score *=kernel_range/score_range
score = score - score[2] + segment_mean[2]
plt.plot([0,1,2,3,4,5],score,color='black',linestyle=":")

ax = sns.tsplot(time="segment", 
                value="norm_stretch", 
                err_style=['ci_band','unit_traces'], 
                condition = 'musician', 
                unit='subj',
                data=kernels, 
                ci=95)
plt.xlabel("Time")
plt.ylabel("Stretch")

plt.subplot(122)
#plt.plot([0,20],[score[2],score[2]],color='black',linestyle=":")
segments = kernels.groupby(['segment','subj'])['norm_stretch','musician','melodie','rythme','rythme-melodie'].first().reset_index()
segments_2 = segments[segments.segment==2]
segments_4 = segments[segments.segment==4]
downbeats = pd.merge(segments_2, segments_4, on=["subj"])
downbeats['downbeat']=downbeats['norm_stretch_x'] / downbeats['norm_stretch_y']
downbeats
sns.regplot(data=downbeats,
            x='rythme-melodie_x',
            #x='musician_x',
            y='norm_stretch_y', 
            color = np.array([65.,105.,225.])/255, label ='note 5')
sns.regplot(data=downbeats,
            x='rythme-melodie_x',
            y='norm_stretch_x',
            color = 'orange', label = 'note 3')
plt.xlabel("Musical skills")
plt.ylabel("")
plt.legend()
_=plt.xlim([4,18])
```

In [15]:

```
from rpy2.robjects import r,pandas2ri
pandas2ri.activate()
from rpy2.robjects.packages import importr
base = importr('base')
ez = importr('ez')
df=pandas2ri.py2ri(kernels[~(kernels['subj'].isin([5,6]))])

r_anova = r("""
library(ez)
function(df) {
    segment <- as.ordered(df$segment)
    subject <- as.factor(df$subj)
    norm_stretch <- as.numeric(df$kernel_stretch)
    musician <- as.factor(df$musician)
    data_anova <- data.frame(subject,segment,norm_stretch, musician)
    ezANOVA(
        data=data_anova,
        dv=norm_stretch,
        wid=subject,
        within=segment,
        between=musician,
        detailed=TRUE)
}
""")
print r_anova(df)

# returns multcmp posthocs
r_lme = r("""
library(nlme)
library(multcomp)
function(df) {
    segment <- as.ordered(df$segment)
    subject <- as.factor(df$subj)
    norm_stretch <- as.numeric(df$kernel_stretch)
    musician <- as.factor(df$musician)
    data_anova <- data.frame(subject,segment,norm_stretch, musician)
    Lme.mod <- lme(norm_stretch ~ segment*musician, random = ~1 | subject, data = data_anova)
    #summary(Lme.mod)
    #anova(Lme.mod)
    summary(glht(Lme.mod, linfct=mcp(segment = "Tukey"), test = adjusted(type = "bonferroni")))
}
""")
print r_lme(df)
```

```
$ANOVA

            Effect DFn DFd         SSn        SSd          F            p p<.05

1      (Intercept)   1   8 0.410244898 0.01113207 294.820121 1.346235e-07     *

2         musician   1   8 0.002087415 0.01113207   1.500109 2.554923e-01      

3          segment   5  40 0.291281312 0.07579277  30.745025 1.052423e-12     *

4 musician:segment   5  40 0.023858257 0.07579277   2.518262 4.500104e-02     *

         ges

1 0.82516064

2 0.02345087

3 0.77016545

4 0.21536008


$`Mauchly's Test for Sphericity`

            Effect          W         p p<.05

3          segment 0.04390813 0.1920944      

4 musician:segment 0.04390813 0.1920944      


$`Sphericity Corrections`

            Effect       GGe        p[GG] p[GG]<.05      HFe        p[HF]

3          segment 0.6149905 1.496813e-08         * 1.042612 1.052423e-12

4 musician:segment 0.6149905 8.024732e-02           1.042612 4.500104e-02

  p[HF]<.05

3         *

4         *


	 Simultaneous Tests for General Linear Hypotheses


Multiple Comparisons of Means: Tukey Contrasts


Fit: lme.formula(fixed = norm_stretch ~ segment * musician, data = data_anova, 

    random = ~1 | subject)


Linear Hypotheses:

           Estimate Std. Error z value Pr(>|z|)    

1 - 0 == 0 -0.03981    0.02457  -1.620  0.58513    

2 - 0 == 0  0.12950    0.02457   5.271  < 0.001 ***

3 - 0 == 0  0.06849    0.02457   2.787  0.05945 .  

4 - 0 == 0  0.14383    0.02457   5.854  < 0.001 ***

5 - 0 == 0  0.05067    0.02457   2.062  0.30728    

2 - 1 == 0  0.16931    0.02457   6.891  < 0.001 ***

3 - 1 == 0  0.10829    0.02457   4.408  < 0.001 ***

4 - 1 == 0  0.18364    0.02457   7.474  < 0.001 ***

5 - 1 == 0  0.09048    0.02457   3.683  0.00306 ** 

3 - 2 == 0 -0.06101    0.02457  -2.483  0.12894    

4 - 2 == 0  0.01433    0.02457   0.583  0.99215    

5 - 2 == 0 -0.07883    0.02457  -3.208  0.01669 *  

4 - 3 == 0  0.07535    0.02457   3.067  0.02642 *  

5 - 3 == 0 -0.01782    0.02457  -0.725  0.97893    

5 - 4 == 0 -0.09316    0.02457  -3.792  0.00203 ** 

---

Signif. codes:  0 '***' 0.001 '**' 0.01 '*' 0.05 '.' 0.1 ' ' 1

(Adjusted p values reported -- single-step method)
```
